# Androgens Regulate T47D Cells Motility and Invasion through Actin Cytoskeleton Remodeling

**DOI:** 10.3389/fendo.2016.00136

**Published:** 2016-09-30

**Authors:** Maria Magdalena Montt-Guevara, Jorge Eduardo Shortrede, Maria Silvia Giretti, Andrea Giannini, Paolo Mannella, Eleonora Russo, Alessandro David Genazzani, Tommaso Simoncini

**Affiliations:** ^1^Molecular and Cellular Gynecological Endocrinology Laboratory (MCGEL), Department of Clinical and Experimental Medicine, University of Pisa, Pisa, Italy; ^2^Department of Obstetrics and Gynecology, Center for Gynecological Endocrinology, University of Modena and Reggio Emilia, Modena, Italy

**Keywords:** androgens, breast cancer, metastasis, actin cytoskeleton, Moesin

## Abstract

The relationship between androgens and breast cancer is controversial. Androgens have complex effects on breast cancer progression and metastasis. Moreover, androgen receptor (AR) is expressed in approximately 70 to 90% of invasive breast carcinomas, which has prognostic relevance in basal-like cancers and in triple-negative breast cancers. Recent studies have associated the actin-binding proteins of the ezrin–radixin–moesin (ERM) family with metastasis in endocrine-sensitive cancers. We studied on T47D breast cancer cells whether androgens with different characteristics, such as testosterone (T), dihydrotestosterone (DHT), and dehydroepiandrosterone (DHEA) may regulate breast cancer cell motility and invasion through the control of actin remodeling. We demonstrate that androgens promote migration and invasion in T47D *via* Moesin activation. We show that T and DHEA exert their actions *via* the AR and estrogen receptor (ER), while the non-aromatizable androgen – DHT – only recruits AR. We further report that androgen induced significant changes in actin organization with pseudopodia along with membrane ruffles formation, and this process is mediated by Moesin. Our work identifies novel mechanisms of action of androgens on breast cancer cells. Through the modulation of Moesin, androgens alter the architecture of cytoskeleton in T47D breast cancer cell and promote cell migration and invasion. These results could help to understand the biological actions of androgens on breast cancer and, eventually, to develop new strategies for breast cancer treatment.

## Introduction

Breast cancer is the most commonly diagnosed cancer in women. About one out of eight women develop breast cancer throughout life (1). Early detection through screening programs and new therapeutic strategies have improved the chances to survive; however, many women still die because of metastasis.

Steroid hormones are the major modulators of breast cancer development and progression. In particularly, androgens and androgen receptors (ARs) have complex effects on breast cancer progression and metastasis (2). The available information on how androgens modulate breast cancer cells behavior is contradictory (3). Most reports indicate that androgens decrease proliferation of breast cancer cell lines (4). Mechanisms such as the inhibition of ERα transactivation activity support such effects (5). In agreement, women receiving androgens have a lower risk of developing breast cancer (6).

However, AR is expressed in approximately 70–90% of invasive breast carcinomas, which has prognostic relevance in basal-like cancers and in triple-negative breast cancers (7, 8). Moreover, AR overexpression is associated with acquisition of resistance to tamoxifen (9) and aromatase inhibitors (10, 11), which is key for cancer progression. Moreover, epidemiological evidence indicates variable associations between concentrations of testosterone (T), androstenedione, dehydroepiandrosterone (DHEA), or sex hormone-binding globulin with breast cancer risk (12, 13).

Recent work in breast cancer cells shows that signaling cascades linked to actin cytoskeleton remodeling, cell motility, and invasion are activated by androgens (14, 15). Cell migration is an integrated process requiring the development of a leading edge (at the front) and of a trailing edge (at the back). The cell’s front is a site of rapid actin polymerization: this pushes the leading front forward and leads to the formation of specialized membrane structures called ruffles, pseudopodia, and lamellipodia, where interactions of the cell with other cells or with extracellular proteins are made possible (16). One of the main sets of controllers in this process is the ezrin–radixin–moesin (ERM) family of actin-binding proteins. The active forms of these proteins bind fibrillar actin, inducing its de-polymerization and its localization to the cell membrane to form sub-membrane complexes (17).

We studied whether androgens with different characteristics, such as testosterone (T), dihydrotestosterone (DHT), and DHEA may regulate breast cancer cell motility and invasion through the control of actin remodeling. We also tested if such actions are exerted through the modulation of the actin-binding protein, Moesin.

## Materials and Methods

### Cell Cultures and Treatments

#### Cell Culture

Human breast carcinoma cell line T47D was obtained from American Type Culture Collection. T47D cells were grown in RPMI-1640 supplemented with 10% FBS (Gibco, Invitrogen), 2 mM l-Glutamax (Gibco, Invitrogen), and ATB (Gibco, Invitrogen). Before experiments, medium were replaced for 24 h with steroid-deprived FBS (Lonza Walkersville, Inc.), and whenever experiments investigated non-genomic effects, cells were kept in medium containing non-FBS for 8 h. Every time an inhibitor was used, the active treatments were done 30 min afterward it. Control cells always received the same amount of ethanol (solvent for E2 or androgens, 0.01% final concentration).

#### Treatments

Testosterone, DHT, DHEA (10^−9^–10^−7^M), 17β-estradiol (E2, 10^−9^M), flutamide (FLUT, 10^−6^M), aminoglutethimide (AG, 10^−6^M), pertussis toxin (PTX, 100 ng/mL), and Rho-kinase inhibitor Y-27632 (RKI, 10^−5^M) were all purchased from Sigma-Aldrich USA. ICI 182,780 (ICI, 10^−6^M) was obtained from Tocris Cookson, UK.

### Immunoblottings

After treatments, cells were collected on ice with lysis buffer containing 50 mM Tris–HCl, pH 7.4, 1 mM EDTA, 1% IGEPAL, protease inhibitor cocktail (Sigma-Aldrich, USA), and phosphatase inhibitor cocktail 3 (Sigma-Aldrich, USA). The concentration of total proteins was quantified by Pierce Micro BCA Assay (Thermo Fisher Scientific). Samples, containing 25 μg of protein, were separated on 10% SDS-PAGE gels and transferred to a PVDF membrane (Immobilon-P, Millipore). Antibodies against the following proteins were used: Moesin (sc-610402), Thr^558^-p-Moesin (sc-12895), AR (sc-816), estrogen receptor (ER) (sc-8005), and GAPDH (sc-59540), all purchased from Santa Cruz Biotechnology. Primary and secondary antibodies were incubated with standard technique. Immunodetection was accomplished with a quantitative digital imaging system (Quantity One; BioRad, USA). Densitometric analysis of the proteins bands was performed using the NIH ImageJ 1.49p software.

### Cell Immunofluorescence

T47D cells were grown on coverslips and exposed to treatments. Cells were fixed with methanol at −20°C for 10 min. Blocking was performed with 3% serum for 20 min. Cells were incubated with Texas Red-X Phalloidin (Sigma). Nuclei were counterstained with 4′-6-diamidino-2-phenylindole DAPI (Sigma) and mounted with VectaShield mounting medium (Vector Laboratories, Burlingame, CA, USA). Inmunofluorescencia was visualized using an Olympus BX41 microscope and recorded with a DP70 Olympus digital camera. The red values of the 40 cells per condition were quantified using the NIH ImageJ 1.49p software by measuring 10 μM distances encompassing the extracellular area, the full thickness of the membrane, and the intracellular space. Two separate measures were taken in each cell. Each experiment was repeated three times.

### Gene Silencing with RNA Interference

Gene silencing was performed using synthetic small interfering RNAs (siRNAs) targeting Moesin, ERα, and AR, purchased from Santa Cruz Biotechnology: sc-35955, sc-29305, sc-29204, respectively. All siRNAs were used at a final concentration of 50–75 nM according to the manufacturer’s instructions. T47D were transfected with Lipofectamine RNAiMAX (Invitrogen, USA) in opti-MEM without ATB. Efficacy of gene silencing was checked after 24, 48, and 72 h by western analysis and found to be optimal from 24 h with 75 nMoL. T47D cells were treated 24 h after siRNA transfection. Each experiment was repeated three times.

### Cell Migration Assay

Cell migration was assayed with razor scrape assays. Briefly, a razor blade was pressed through the confluent T47D breast cancer cell monolayer into the plastic plate to mark the starting line. T47D cells were swept away on one side of that line. Cytosine β-d-arabinofuranoside hydrochloride (Sigma) (10 μM), a selective inhibitor of DNA synthesis which does not inhibit RNA synthesis, was added 1 h before the treatments. Absence of cell proliferation was checked in preliminary experiments with MTT (3-(4, 5-dimethylthiazol-2-yl)-2, 5-diphenyltetrazolium bromide) assay. Migration was monitored for 48 h. Every 24 h, fresh mediums and treatments were replaced. The migration area was visualized using an Olympus BX41 microscope and recorded with a high-resolution DP70 Olympus digital camera. The migration area was measurement from four different fields under 40× magnifications for each condition. Each experiment was repeated three times.

### Cell Invasion Assay

Cell invasion was assayed using the BD BioCoat Growth Factor Reduced (GFR) Matrigel Invasion Chamber (BD Bioscience, USA). In brief, after rehydrating the GFR Matrigel inserts, the test substance was added to the wells. An equal number of Control Inserts (no GFR Matrigel coating) was used. The chambers were incubated for 24 h at 37°C, 5% CO_2_ atmosphere. After incubation, non-invading cells were removed from the upper surface of the membrane using cotton swabs. Then the cells on the lower surface of the membrane were stained with Diff-Quick. Invading cells were visualized using a 60× magnification Olympus BX41 microscope and photographed with a high-resolution DP70 Olympus digital camera. The invasion value was quantified from the mean number of five different fields per condition. The normalized values were then used to calculate the Invasion index. Each experiment was repeated three times.

### Statistical Analysis

Each experimental condition was reproduced in three independent experiments. All data are presented as mean ± SEM. Statistical analysis was performed using GraphPad Prism 6 (GraphPad Software). Statistical differences between means values were analyzed using one-way anova followed by Bonferroni posttest. Differences at *p* < 0.05 were considered significant.

## Results

### Androgen Administration to T47D Cells Enhances Cell Migration and Invasion

We, first, studied whether androgen administration to T47D (AR+/ER+) breast cancer cells turns into modulation of cell migration. We used three different androgens that have physiological and clinical relevance: the aromatizable androgen testosterone (T), the non-aromatizable testosterone metabolite, such as DHT, and the androgenic/estrogenic precursor, such as DHEA. The three androgens were tested at different concentrations spanning from physiological (10^−9^–10^−7^M) for 48 h. To eliminate effects on cell proliferation, T47D cells were treated with cytosine arabinoside (1-β-d-arabino-furanosyl)-cytosine hydrochloride (Ara-C, 10^−5^M), an inhibitor of DNA synthesis that prevents cell division, but allows RNA synthesis.

**Figure 1:**
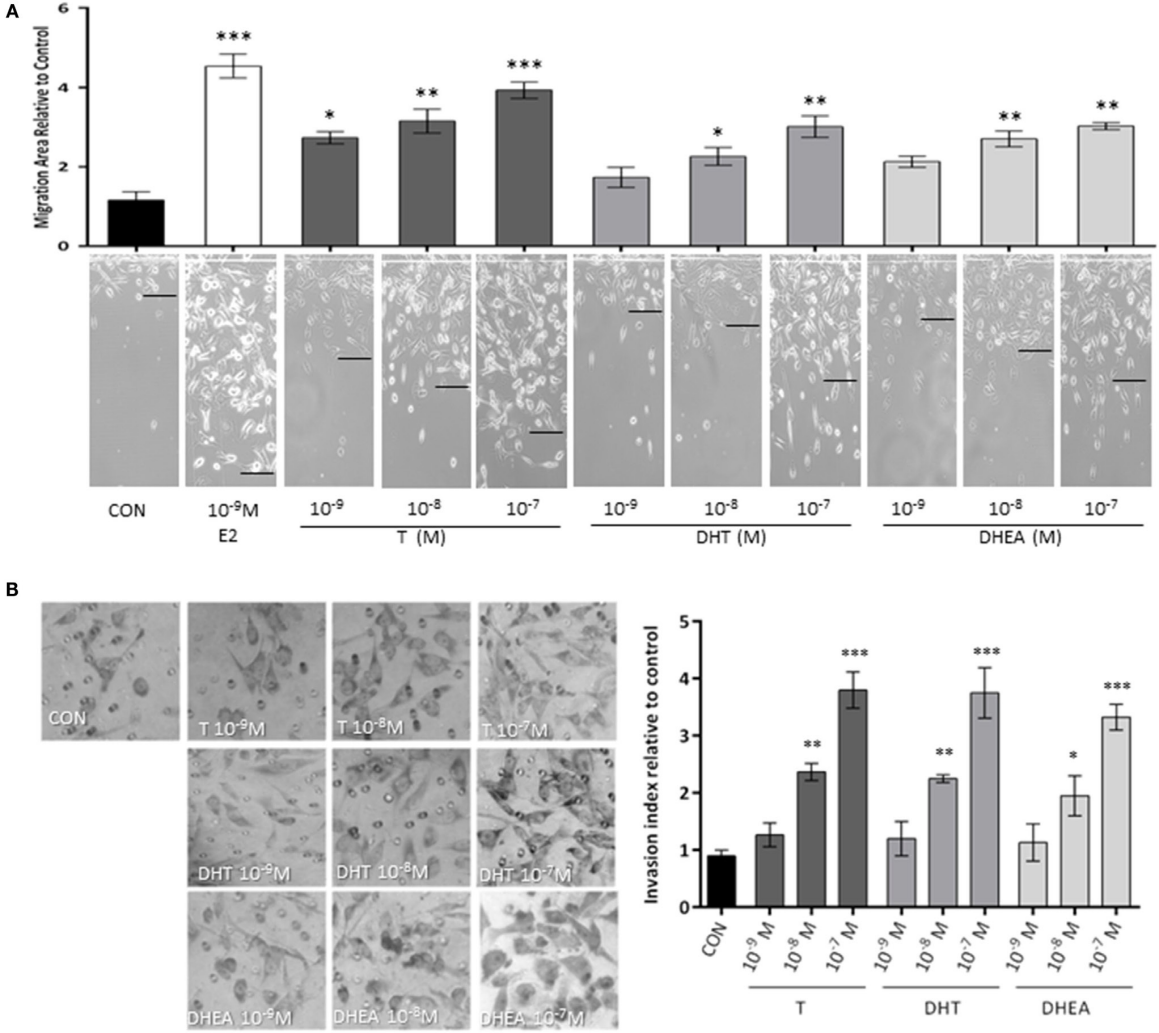
**Androgens enhance T47D breast cancer cell migration and invasion**. Steroid-deprived, ER/AR+ T47D cells were treated with 10^−9^M E2 or with increasing concentrations of T, DHT, and DHEA (10^−9^M–10^−7^M). **(A)** Cell migration was assayed with razor scrape assays. Horizontal cell migration was measured as the mean number of cells crossing the starting line after 48 h of each treatment. **(B)** Cell T47D invasion was assayed with matrigel invasion chambers; after 24 h, invasion index was quantified from the mean number of invading cells in the membrane. Images are representative and the bar graph show the mean ± SEM of the migration area and invasion index relative to control of three independent experiments (*p ≤ 0.05, **p ≤ 0.01, ***p ≤ 0.001 versus control).

Exposure to T (10^−9^–10^−7^M) significantly increased T47D cell migration (Figure 1A), while with DHT and DHEA, we observed an increase only at higher concentrations (10^−8^ and 10^−7^M). Cell migration was related to the concentration of androgen provided. As a comparator, the extent of migration obtained with the higher dose of androgens was similar to that achieved with a physiological amount of E2 (10^−9^M).

Then, we tested the effects of the same androgens on T47D cell invasion. Cells displayed a significant increase of invasive behavior when exposed to 10^−8^M and 10^−7^M of all androgens, which correspond to the high physiological range for T and DHEA and around 10-fold higher than the normal concentration in adults for DHT (Figure 1B).

### Androgens Induce Rapid Cytoskeletal Rearrangements and the Development of Specialized Membrane Structures

**Figure 2:**
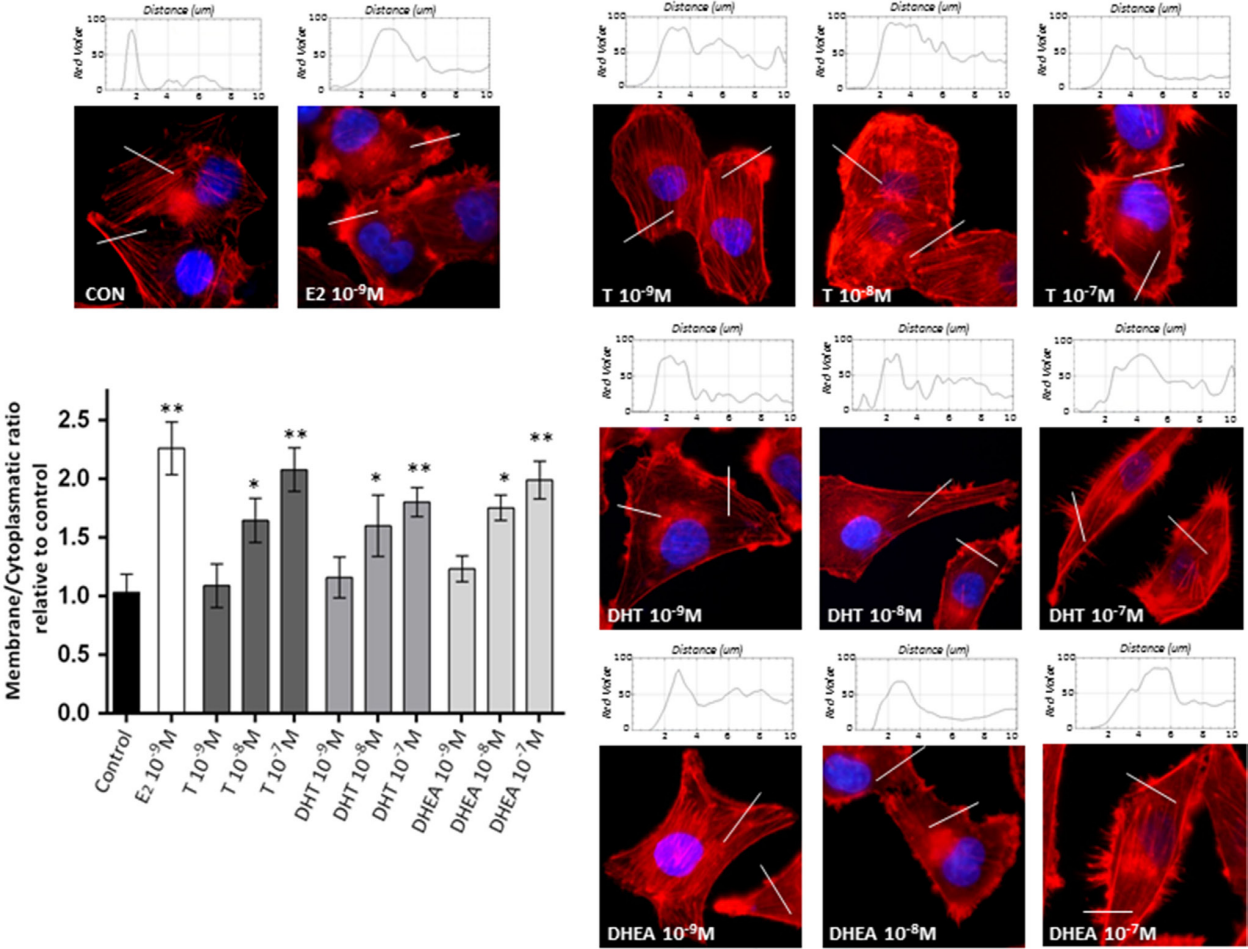
**Androgens trigger actin cytoskeleton and cell membrane remodeling in T47D cells**. T47D cells were treated with E2 (10^−9^M) or with increasing concentrations of T, DHT, or DHEA (10^−9^M–10^−7^M) for 20 min. Actin filaments were stained with phalloidin linked to Texas Red (red staining), and nuclei were counterstained with DAPI (blue staining). Immunofluorescence analysis reveals the dynamic modifications of actin filaments localization and the formation of specialized cell membrane structures. The histograms represent the quantification of fluorescence intensity across the lines of 40 cells of each group using ImageJ software. Images are representative and the bar graph represents the mean ± SEM of the membrane/cytosol actin fluorescent intensity ratio relative to control of triplicate experiments (*p ≤ 0.05, **p ≤ 0.01 versus control).

To assess if the effects of androgens on breast cancer cell invasion are linked to modifications of the actin cytoskeleton, we stained actin filaments with Phalloidin-Texas Red and used fluorescence microscopy to visualize actin filaments. Non-treated cells displayed mainly longitudinally arranged actin filaments in the cytoplasm (Figure 2). However, when cells were exposed to T, DHT, and DHEA (10^−9^–10^−7^M), we observed a rapid change in actin organization, with a remodeling of the filaments toward the cell membrane edge after 20 min of treatment. Significant changes in actin organization with pseudopodia and membrane ruffles formation became visible from 10^−8^M for all androgens assayed (Figure 2). As we have shown in previous report (18, 19), E2 10^−9^M was used as positive control of actin filaments remodeling.

### T, DHT, and DHEA Activate the Actin-Regulatory Protein, Moesin, in T47D Cells

To study if the modifications in actin arrangement and the parallel enhancement of T47D cell motility may be linked to regulation of actin-binding proteins, we analyzed if the ERM family member, Moesin, is functionally activated during exposure to androgens (20, 21).

**Figure 3:**
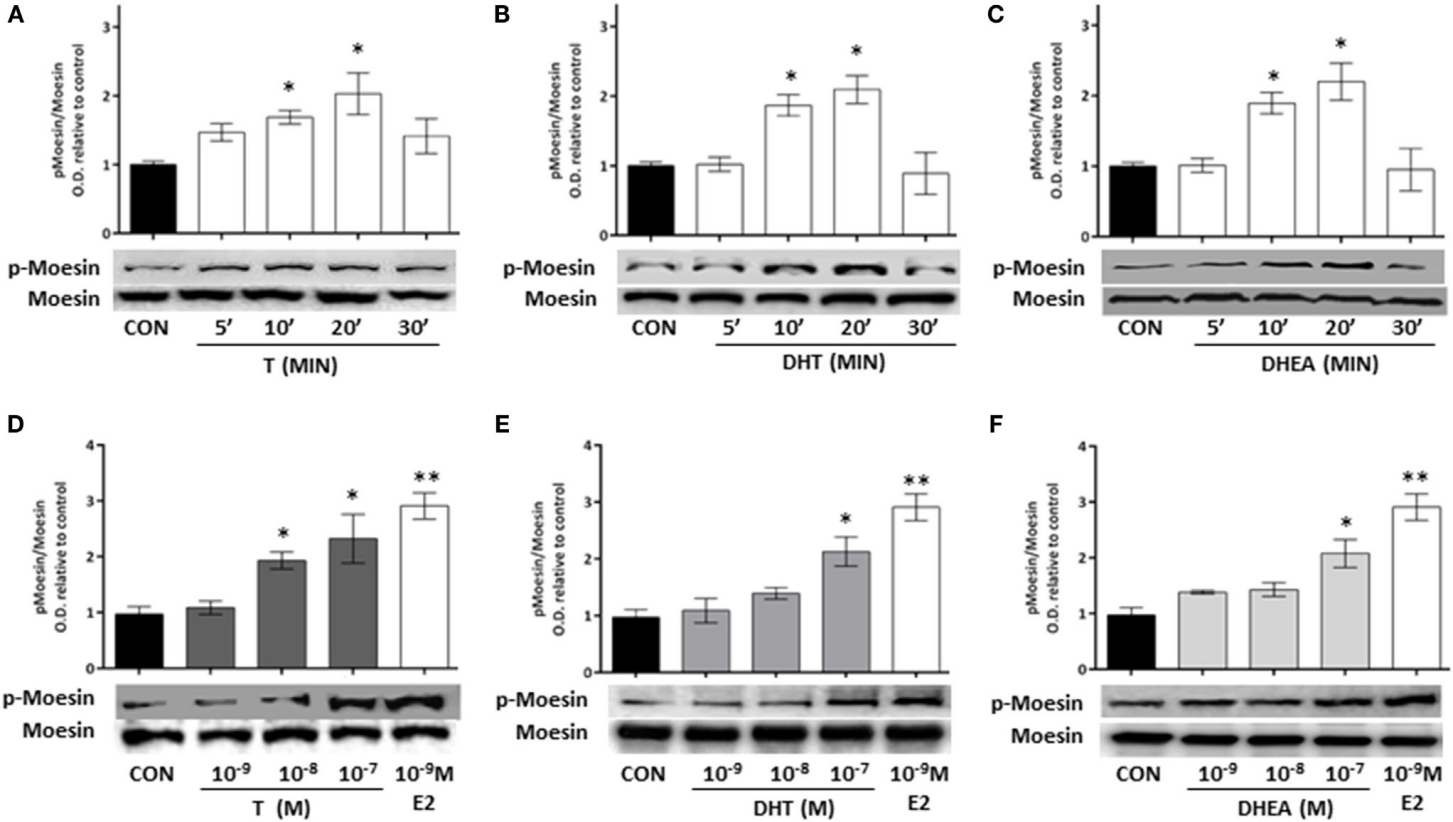
**Androgen administration to T47D cells results in Moesin activation**. **(A–C)** T47D cells were treated for different times (5, 10, 20, and 30 min) with 10^−8^M T, DHT, and DHEA. **(D–F)** T47D cells were treated for 20 min with increasing concentrations of T, DHT, and DHEA (10^−9^M–10^−7^M), E2 was used as control. Whole cell extracts were resolved by SDS-PAGE, and wild-type Moesin and Thr^558^-phosphorylated Moesin (p-Moesin) levels were analyzed by western blot. Images are representative of triplicate experiments. Representative images are shown, and the bar graph show the mean ± SEM of the ratio Moesin/p-Moesin optical density relative to control of three independent experiments (*p ≤ 0.05, **p ≤ 0.01, ***p ≤ 0.001 versus control).

Cell treatment with T, DHT, and DHEA turned into a rapid increase of Thr^558^ phosphorylation of Moesin (Figures 3A–C). Intensity of Moesin phosphorylation was related to the concentration of T, DHT, and DHEA (Figures 3D–F). A significant increase of Moesin phosphorylation was observed from 10^−8^M for T and 10^−7^M for DHT and DHEA. Parallel experiments showed that physiological concentrations of E2 induce Moesin activation (Figures 3D–F).

### Moesin Is Required for the Androgen-Induced Increase in T47D Cell Migration

**Figure 4:**
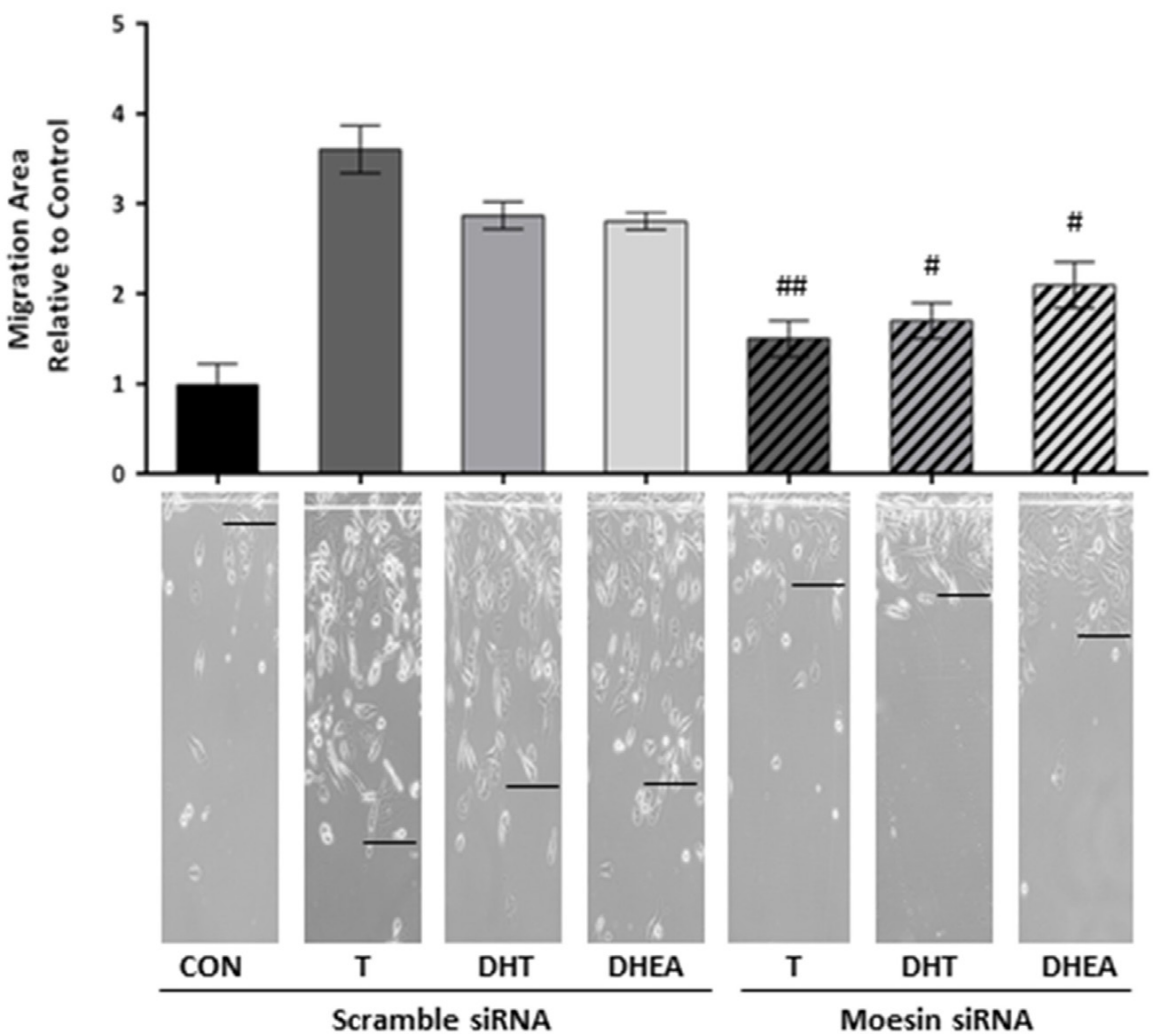
**Moesin is required for androgens-induced T47D cell migration**. T47D cells were transfected with Moesin siRNAs 24 h before performing the horizontal migration assay. Breast cancer cells were scraped out of the cell culture dish with a razor blade at the beginning of the experiment and treated with 10^−7^M T, DHT, or DHEA for 48 h. Horizontal cell migration was measured as the number of cells crossing the starting line. Images are representative, and the bar graph shows the mean ± SEM of the migration area relative to control of three independent experiments (^#^*p* ≤ 0.05, ^##^*p* ≤ 0.01, ^###^*p* ≤ 0.001 versus each treatment with Scramble siRNA).

To corroborate whether Moesin is required for androgen-dependent activation of T47D cell motility, we downregulate Moesin expression with siRNAs. Moesin silencing led to a significant decrease in cell migration induced by all androgens (Figure 4), while treatment with an inactive, scrambled RNA did not affect pro-migratory androgen effects (Figure 4).

### Androgens Activate Moesin and Actin Remodeling through Androgen and Estrogen Receptors

To study the signaling pathways involved in the effects of T, DHT, and DHEA, we used a set of pharmacological inhibitors including: the AR antagonist FLUT, the ER antagonist ICI 182,780 (ICI), the aromatase inhibitor AG, the G protein inhibitor PTX, and the RKI.

**Figure 5:**
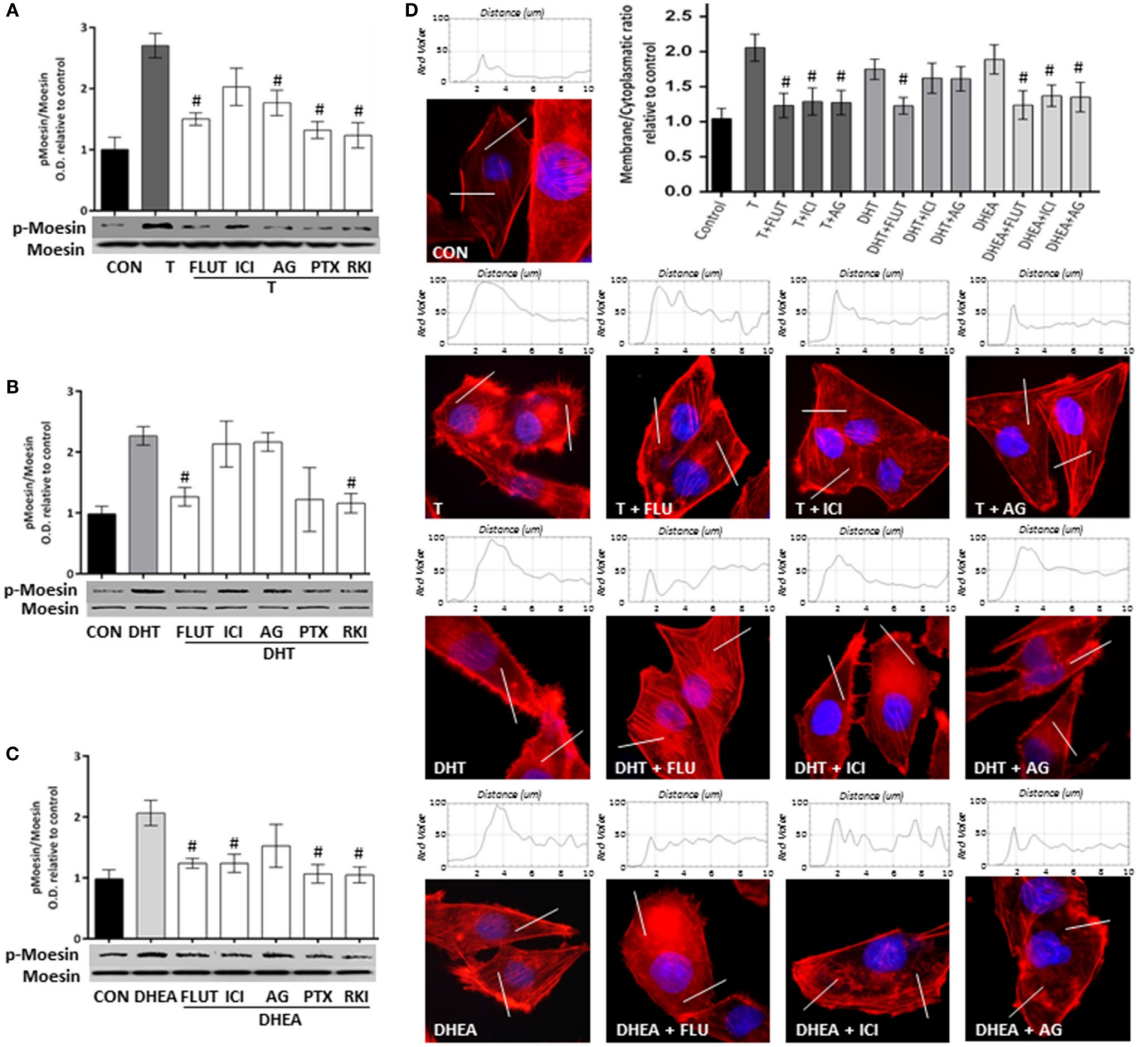
**Androgens activate Moesin and actin remodeling through androgen and estrogen receptors in T47D cells**. T47D cells were treated with 10^−7^M T, DHT, or DHEA for 20 min, in the presence or absence of the AR inhibitor flutamide (FLUT, 10^−6^M), the ER antagonist ICI 182,780 (ICI, 10^−6^M), the aromatase inhibitor aminoglutethimide (AG, 10^−6^M), the G protein inhibitor pertussis toxin (PTX, 100 ng/mL), and the Rho-kinase inhibitor Y-27632 (RKI, 10^−5^M). **(A–C)** Whole cell extracts were resolved by SDS-PAGE and wild-type Moesin, and Thr^558^-phosphorylated Moesin (p-Moesin) levels were analyzed by western blot. Images are representative of triplicate experiments. Representative images are shown, and the bar graph shows the mean ± SEM of the ratio Moesin/p-Moesin optical density relative to control of three independent experiments (^#^*p* ≤ 0.05 versus each treatment). **(D)** Actin filaments were stained with phalloidin linked to Texas Red (red staining), and nuclei were counterstained with DAPI (blue staining). Immunofluorescence analysis reveals the dynamic modifications of actin fibbers localization and the formation of specialized cell membrane structures. The box on top of the cells display the intensity of the signal throughout the sample areas measure (two per cell, indicated as the white line). Images are representative, and the bar graph represents the mean ± SEM of the membrane/cytosol actin fluorescent intensity ratio relative to control of triplicate experiments (^#^*p* ≤ 0.05 versus each treatment).

Flutamide and AG decreased significantly Moesin phosphorylation induced by T, whereas ICI was less effective, suggesting that T acts though both AR and ER (Figure 5A). Phosphorylation of Moesin induced by DHT was significantly decreased by FLUT, but not by ICI or AG (Figure 5B). Because DHT cannot be aromatized to estrogen metabolites or does not directly bind ER, this result confirms that it can only act *via* AR. Moesin activation induced by DHEA was significantly reduced by FLUT and ICI, but was counteracted by AG (Figure 5C). This indicates that DHEA, which can be metabolized to estrogenic and androgenic metabolites, may act particularly through ER.

A similar pattern of pharmacological inhibition was seen when FLUT, ICI, or AG were used to counteract the actions of the three androgens on actin cytoskeleton remodeling (Figure 5D).

Pertussis toxin and PKI inhibited Moesin phosphorylation induced by androgens (Figures 5A–C), suggesting that a G protein- and ROCK-2-dependent signaling cascade is necessary. This is consistent with our previous reports where steroid hormone receptors activate G-protein–ROCK-2-Moesin signaling in breast cancer and other cell models (22–25).

To corroborate this findings, we performed the same experiments in MCF7, ER (+), AR (+) and in MDA-MB231, ER (−), AR (−). We found that FLUT, AG, and RKI, in presence of T, significantly decrease Moesin phosphorylation in MCF7 cells. On the other hand, Moesin activation by DHT was reduced when MCF7 were treated with FLUT, PTX, and RKI (Figure S1 in Supplementary Material). Furthermore, DHEA activity was diminished by all inhibitors, suggesting that ER and AR are involved in Moesin activation. However, when MDA-MB231 cells were treated with T, DHT, and DHEA, we saw no significant differences in the levels of phosphorylated Moesin (p-Moesin) (Figure S1 in Supplementary Material).

### Androgens Stimulate Cell Invasion *via* Moesin Activation through ERα and AR

**Figure 6:**
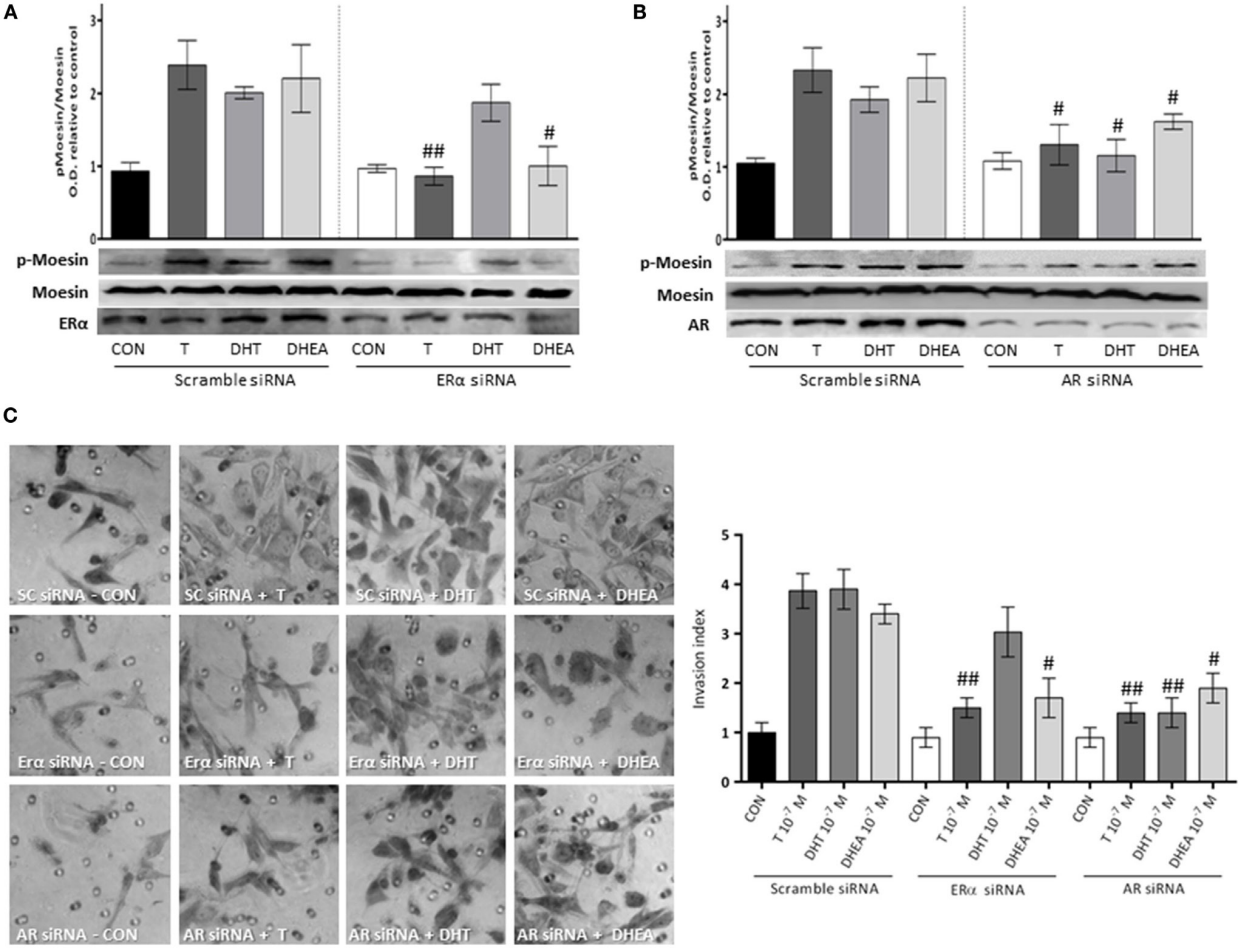
**Androgens activation of Moesin and T47D cell invasion: relative contribution of ER and AR**. T47D cells were transfected with ERα, AR, and Scramble siRNAs 24 h before experiments were performed. **(A,B)** Transfected Cells were treated for 20 min with 10^−7^M T, DHT, or DHEA, and protein extracts were obtained. Western blot for total Moesin, p-Moesin, ERα, and AR were done. Images are representative of triplicate experiments. Representative images are shown, and the bar graph shows the mean ± SEM of the ratio Moesin/p-Moesin optical density relative to control of three independent experiments (^#^*p* ≤ 0.05, ^##^*p* ≤ 0.01 versus each treatment with Scramble siRNA). **(C)** Transfected cells were treated with 10^−7^M T, DHT, or DHEA, and cell invasion was assayed with matrigel invasion chambers for 24 h. Invasion index was quantified from the mean number of invading cells in the membrane. Images are representative, and the bar graph shows the mean ± SEM of invasion index relative to control of three independent experiments (^#^*p* ≤ 0.05, ^##^*p* ≤ 0.01 versus each treatment with Scramble siRNA).

To corroborate the hypothesis that Moesin activation by androgens happens upon activation of either ERα or AR, we downregulated both receptors with siRNAs. We observed a significant decrease in Moesin phosphorylation when ERα was downregulated in cells treated with T and DHEA, but not with DHT (Figure 6A). On the other hand, downregulation of AR affected significantly Moesin activation associated with T, DHT, and DHEA treatments (Figure 6B).

Consistent with these results, the pro-invasive effects of T and DHEA were significantly decreased in cells where AR and ERα were downregulated (Figure 6C). Conversely, the pro-invasive effect of DHT in 3-dimensional matrices was significantly decreased in cells where AR expression was silencing (Figure 6C).

## Discussion

The main finding of this study is that androgens modulate actin cytoskeleton rearrangement in T47D cells, thereby influencing cell migration and invasion. A precise regulation of actin dynamics is necessary for cell migration, which is required for cancer spread, invasion, and metastasis (18).

Metastasis is a complex multistep process that involves protrusion of the leading edge of the cell, formation of adhesion complexes, myosin/actin-mediated cell contraction, and the release of adhesions at the cell rear (26). One of the major players that participate in this process is the actin-binding protein Moesin. Moesin and its parent Radixin have been associated with high metastatic potential in the clinical setting. For instance, overexpression Moesin and Radixin are present in pancreatic cancers with lymph node metastases as compared with those where metastases are absent (27). Moesin seems also to be an important promoter of epithelial–mesenchymal transition (EMT) in human mammary cell MCF10A, and it is highly expressed in a variety of human cancers and cancer cell lines (28–30). Moesin is regulated by many different stimuli, and steroid hormones and their receptors seem to be particularly important (22, 23, 25).

Androgen receptor is expressed in approximately 70 to 90% of invasive breast carcinomas, which has prognostic relevance in basal-like cancers and in triple-negative breast cancers (7), suggesting that it could have a biological relevance in the course of the disease. Historical reports identify androgens as hormones that blunt cancer cells proliferation in the breast (2, 31). But, more recent clinical evidence associates overexpression of AR with loss of sensitivity to standard endocrine adjuvant therapies (10, 32), hence increased cancer aggressiveness. Parallel reports show that AR regulates actin cytoskeleton architecture (14), E-Cadherin expression, EMT, and tumor metastasis in several breast cancer cell lines (33).

Our findings, therefore, raise the issue that androgens could play an important role in cell migration and invasion, processes that are closely related with tumor metastasis. The identification of signaling intermediates with pro-metastatic action regulated by androgens may allow development of therapies that could involve AR and its downstream intermediates as targets. AR inhibitors, in combination with other systemic agents, could be a valuable treatment for a large proportion of breast cancers (34).

From a clinical point of view, the major area of discussion is whether androgen treatments are safe for breast in women, particularly since androgen administration to women has gained momentum in the recent past to counteract decline of libido and wellbeing after the menopause (35).

The relationship between androgens and the breast is largely unclear. This is in part due to the androgens conversion to estrogens through the aromatase system. Some studies suggest that endogenous or exogenous androgens may increase the risk of developing breast cancer, but a vast amount of evidence seems to contradict this view (2, 34, 36). Indeed, women receiving androgens are at lower risk of developing breast cancer (6, 37), and the addition of androgens to tamoxifen increases efficacy to the treatment of advanced breast cancer (38).

Our data shows that T and DHEA exert their effects through both AR and ER, while DHT only recruits AR. This confirms that the biological systems that control breast cancer cell motility and invasiveness are redundantly controlled by different sex steroid receptors. This may be particularly relevant in breast cancer cells, where aromatization of androgens to estrogens is more pronounced compared with normal breast tissue (39).

In conclusion, our work identifies novel mechanisms of action of androgens on breast cancer cells. Through the modulation of the actin-binding protein, Moesin, androgens alter the architecture of the cytoskeleton in T47D breast cancer cells and promote cell migration and invasion. These results help characterizing the biological actions of androgens on breast cancer and, eventually, to develop new strategies for treatment of breast cancer.

## Author Contributions

MM-G, JS, and MG performed the molecular studies and bioinformatics statistical analysis and drafted the manuscript. AG, PM, ER, and ADG participated in the data interpretation and helped to draft the manuscript. TS designed and participated in the data interpretation, helped to draft the manuscript, and supervised the project. All authors read and approved the final manuscript.

## Conflict of Interest Statement

The authors declare that there is no conflict of interest that could be perceived as prejudicing the impartiality of the research reported.

## Supplementary Material

Figure S1 **MCF7 and MDA MB231 cells were treated with 10^−7^M T, DHT, or DHEA for 20 min, in the presence or absence of the AR inhibitor flutamide (FLUT, 10^−6^M), the ER antagonist ICI 182,780 (ICI, 10^−6^M), the aromatase inhibitor aminoglutethimide (AG, 10^−6^M), the G protein inhibitor pertussis toxin (PTX, 100 ng/mL), and the Rho-kinase inhibitor Y-27632 (RKI, 10^−5^M)**. Whole cell extracts were resolved by SDS-PAGE and wild-type Moesin, and Thr^558^-phosphorylated Moesin (p-Moesin) levels were analyzed by western blot. Images are representative of triplicate experiments. Representative images are shown, and the bar graph shows the mean ± SEM of the ratio Moesin/p-Moesin optical density relative to control of three independent experiments (^#^*p* ≤ 0.05 versus each treatment).
