# Supplementary figures and images for: Androgens Regulate T47D Cells Motility and Invasion through Actin Cytoskeleton Remodeling
Source: Front Endocrinol (Lausanne). 2016 Sep 30;7:136. doi: 10.3389/fendo.2016.00136 (PMC5043384; doi:10.3389/fendo.2016.00136)

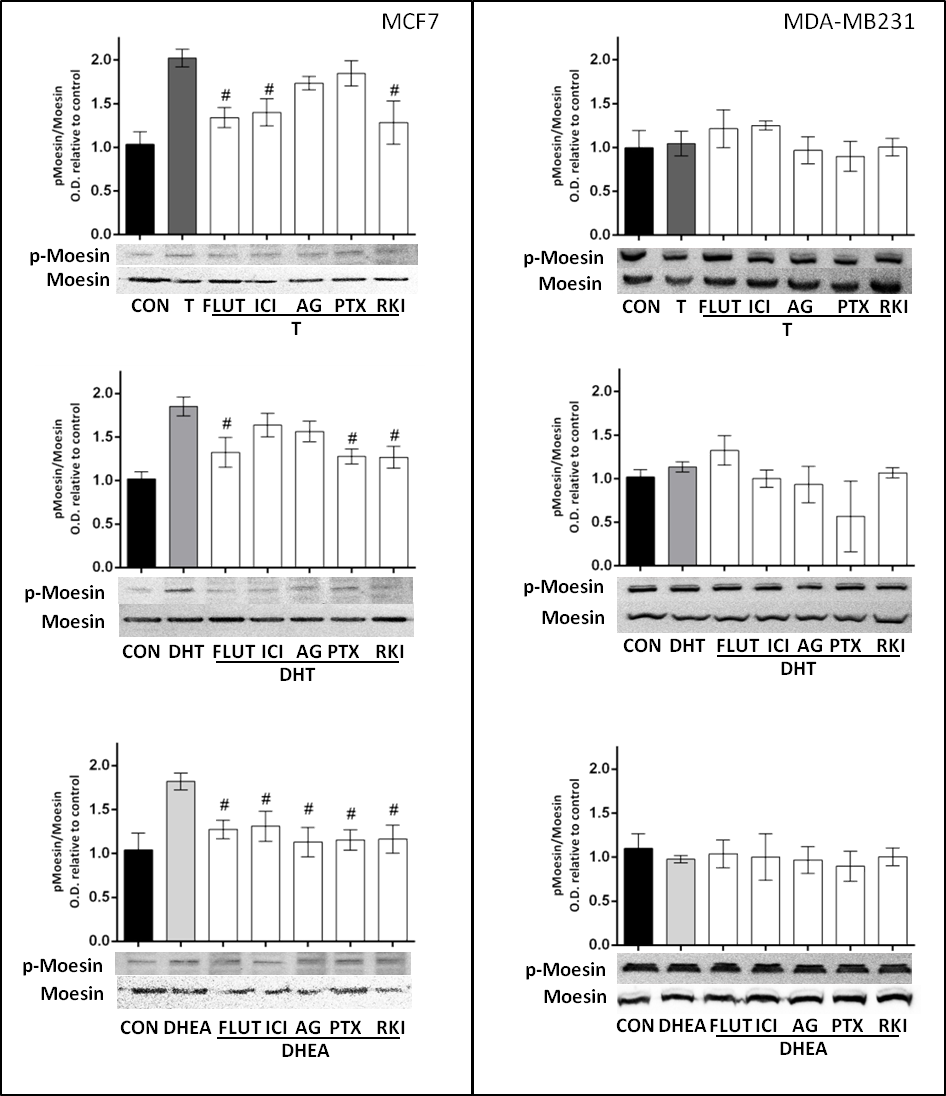

Supplement: Figure S1 — MCF7 and MDA MB231 cells were treated with 10−7M T, DHT, or DHEA for 20 min, in the presence or absence of the AR inhibitor flutamide (FLUT, 10−6M), the ER antagonist ICI 182,780 (ICI, 10−6M), the aromatase inhibitor aminoglutethimide (AG, 10−6M), the G protein inhibitor pertussis toxin (PTX, 100 ng/mL), and the Rho-kinase inhibitor Y-27632 (RKI, 10−5M). Whole cell extracts were resolved by SDS-PAGE and wild-type Moesin, and Thr558-phosphorylated Moesin (p-Moesin) levels were analyzed by western blot. Images are representative of triplicate experiments. Representative images are shown, and the bar graph shows the mean ± SEM of the ratio Moesin/p-Moesin optical density relative to control of three independent experiments (#p ≤ 0.05 versus each treatment). [file image_1.tif]
